# BDHerbalPlants: augmented and curated herbal plants image dataset for classification

**DOI:** 10.1016/j.dib.2025.111885

**Published:** 2025-07-11

**Authors:** Sunzil Khandaker, Md Mizanur Rahman

**Affiliations:** Faculty of Science & Information Technology, Department of CSE, Daffodil International University, Bangladesh

**Keywords:** Herbal plant, Deep learning, Data augmentation, Computer vision, Agricultural informatic, Image classification

## Abstract

This extensive dataset of herbal plants can be highly beneficial for the potential development of agricultural research and practical plant identification tasks. This article introduces a dataset named “BDHerbalPlants” with 1792 raw, high-quality images and 14,336 augmented data images of herbal plants. It contains images of eight distinct herbal plants from different regions, including Dhaka and Tangail. The eight plants are Eclipta prostrata, Ocimum tenuiflorum, Centella asiatica, Mentha arvensis, Kalanchoe pinnata, Azadirachta indica, Coriandrum sativum, Datura stramonium. Each image is carefully captured and labeled after verifying by experts. The significance of this dataset is showcased using popular pre-trained models such as Xception, DenseNet201, and RegNetY032 DL models. This herbal plant data has immense potential to be seamlessly integrated into Deep learning (DL) tasks and play a significant role in the healthcare and pharmaceutical industry. It serves as valuable data for future research for agricultural Informatics and classifying herbal plants that can be found in woods but are challenging to identify without field knowledge

Specifications table

**Table:** 

Subject	Computer Sciences
Specific subject area	Computer Vision, Image classification, Image processing, Machine learning*.*
Type of data	Image.
Data collection	We have collected high quality images over February and March 2025 from Dhaka and Tangail. Images are collected by Poco F5 and Google Pixel 7 smartphones camera. Almost all the pictures have 4624 × 3472/ 3472 × 4624 pixels or 4080 × 3072 pixels*.* The primary goal was to capture leaf and plant body, different sizes of plant were taken as samples with different versions or condition of plants were also captured*.*
Data source location	Institution: National Botanical Garden, Town/City/Region: Zoo Road, Dhaka 1216 Country: Bangladesh Latitude: 24.000° N Longitude: 90.000° E Institution: Kumudini Women’s Medical College, Town/City/Region: Hospital Road Mirzapur, Kumudini Hospital Rd, Mirzapur 1940, Tangail Country: Bangladesh Latitude: 24.0976° N Longitude: 90.0974° E
Data accessibility	Repository name: [Mendeley Data.] Data identification number: [10.17632/md59kt54jy.3] Direct URL to data: [https://data.mendeley.com/datasets/md59kt54jy/3]

## 1 Value of the Data

• The dataset offers a valuable collection of a large number of image data covering eight different types of herbal plants, which are common yet not often recognized for their medicinal value.
• This dataset facilitates the training and evaluation of machine learning and deep learning models for tasks such as object identification, image segmentation, and classification while incurring low computational overhead, hence enabling efficient transfer learning using pre-trained models [1]. Additionally, it supports computer science research and makes it easier to teach taxonomy, plant identification, and the value of medicinal herbs [2].
• It can be helpful in image recognition classifier for herbal plants along with IoT devices to identify them directly from fields or in real-time scenarios with high efficiency, where the plant’s images can help model to train, test, and validate the accurate herbal plant.
• It is available in the public dataset; any interested researcher may access it for free and use it for their research without any cost. It is open for any fellow researcher who wants to contribute to the betterment of human beings*.*

## 2 Background

Herbal plants are naturally abundant and free from synthetic additives, offering remedies for treating common illnesses, boosting immunity, and promoting overall health. These plants are rich in bioactive compounds like coumestans, eugenol, asiaticoside, menthol, bryophyllin, nimbin, linalool, and scopolamine. These compounds empower people to defend against pathogens, mitigate inflammation, treat skin conditions, facilitate wound healing, and enhance the immune system [3]. Yet, in this day and age, we are forgetting about these century-old beneficial herbal medicines, and not many people can identify them now. Therefore, a system is needed to be able to help the community to recognize medicinal plants without difficulty. The motivation behind the study is to make people aware of the many herbal plants surrounding them and classify them, even for those who do not have field knowledge of the plants. The data from eight herbal plants can be used in extensive research of botany tasks as well as plant identification with machine learning, deep learning, and computer vision. As the dataset is publicly available, it is easy and free to access. It holds significance for public awareness and educational campaigns. It can be applied beyond traditional medicine, including agriculture, environmental monitoring, and remote sensing technologies [2].

## 3 Data Description

**Fig. 1:**
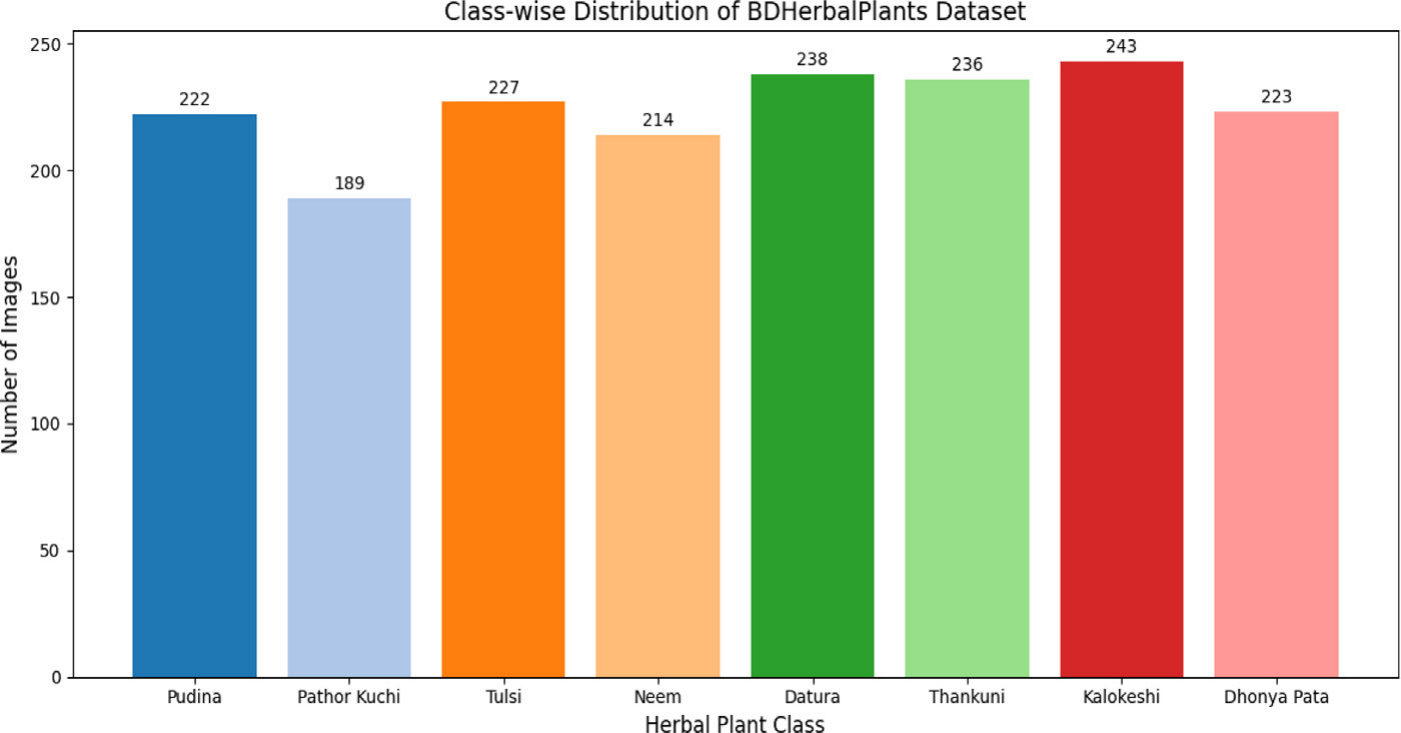
Classwise distribution of BDHerbalPlants Raw Data.

In this new society, information is essential, and everyone should be able to engage everywhere and profit from everything the Information Society has to offer. There are many people who find it too difficult to recognize the correct medicinal plant with the correct medicinal properties because of a lack of knowledge, even when they are living next to it because classifying it is a challenge. This dataset holds eight of these common yet beneficial medicinal herbs for people to classify easily. The “BDHerbalPlants” dataset holds 1792 raw images and 14,336 augmented, pre-processed images of herbal plants [4]. The data are primarily collected from the National Botanical Garden and other data from Kumudini Women’s Medical College. Detailed data distribution of data over the classes is shown in Fig. 1.

The plants include Kalokeshi *(Eclipta prostrata),* Tulsi *(Ocimum tenuiflorum),* Thankuni *(Centella asiatica),* Pudina *(Mentha arvensis),* Pathor Kuchi *(Kalanchoe pinnata),* Neem *(Azadirachta indica),* Dhonya Pata *(Coriandrum sativum) and* Datura *(Datura stramonium).* Each class contains around 200 raw pictures each from 260 different plants. Among them number of distinct plants are Kalokeshi *−40,* Tulsi*-35,* Thankuni*-30,* Pudina*-29,* Pathor Kuchi*-27,* Neem*-32,* Dhonya Pata*-37 and* Datura*-30.*

Data counts are shown in Table 1. The raw images are further got split into 3 folders: train, test and validation. The augmented folder is kept intact for users to use it as they find it best.

**Table 1:** *BDHerbalPlants* plant image categories and counts for raw and augmented data.

Class name	Train	Validation	Test	Total	Augmented images
Datura (Datura stramonium)	166	25	47	238	1904
Neem (Azadirachta indica)	149	23	42	214	1712
Dhonya Pata (Coriandrum Sativum)	156	23	44	223	1784
Pudina (Mentha Arvensis)	155	23	44	222	1776
Tulsi (Ocimum tenuiflorum)	158	24	45	227	1816
Thankuni (Centella asiatica)	165	24	47	236	1888
Pathor Kuchi (Kalanchoe pinnata)	132	20	37	189	1512
Kalokeshi (Eclipta prostrata)	170	25	48	243	1944
Total Images	1251	187	354	1792(Raw)	14,336 (Augmented)

Each of the data class contain healthy data of herbal plants. This will be very beneficial when training deep learning models for plant detection, focusing towards our actual goal. From a better understanding, Table 2 describes the eight specific classes of herbal plants represented in the collected images. Following is the actual class description included in the dataset:

**Table 2:** Overview of herbal plant species.

Name of class	Description	Visualization
**Kalokeshi (Eclipta prostrata)**	A plant belonging to the Compositae family, Eclipta prostrata, has an exceptional reputation in Bangladesh for being used as a medicine. It is well known for glycemic control in diabetes, and no scientific evaluation has been carried out to date to justify its safety and efficacy in glycemic control [5].	
**Tulsi (Ocimum tenuiflorum)**	Tulsi is widely found in tropical Asian wilderness and is well known for its revitalizing and life-extending qualities. Medicinal properties of tulsi are proven against asthma, bronchitis, diabetes, diarrhea, eye conditions (chronic conjunctivitis, cataracts, and glaucoma), fever, insect and snake bites, malaria, and a number of skin conditions [6].	
**Thankuni (Centella asiatica)**	‘Thankuni,’ also known as ‘Goku Kola,’ is popularly used as Thankuni pakora, Thankuni paste, Thankuni juice, or in cooking with fish in Cambodia, Indonesia, Myanmar, Sri Lanka, Thailand, and Vietnam. It is commonly used for human ailments, minor wounds, minor stomach upsets, and diarrhea and some claim it to have properties to improve memory and concentration [7] .	
**Pudina (Mentha arvensis)**	Mentha arvensis, commonly known as Pudina or Mint, is typically found in woods or rocky or cold regions of Bangladesh and India. Treatment of diarrhea. It is likewise utilized to treat liver infection, spleen infection, asthma, and jaundice, while menthol derived from mint is used in drugs, perfumery, and food enterprises [8].	
**Pathor Kuchi (Kalanchoe pinnata)**	Kalanchoe pinnata, also known as cathedral bells, air plant, or Pathorkuchi (in Bangladesh), is native to Madagascar. Has been recorded in Trinidad and Tobago as being used as a traditional treatment for hypertension, also useful against headaches, inflammations, and cancer, and a popular remedy for fevers as well [9].	
**Neem (Azadirachta indica)**	Azadirachta indica has been treated as a medicinal plant since ancient times. Due to its numerous health benefits, many people refer to Azadirachta indica, also known as neem, a tree that originated in India and Myanmar, as “the village pharmacy” or “the divine tree.” Extracts from neem (leaves, bark, fruit, flowers, oil, and gum) have been demonstrated to have a variety of uses, including insect repellent, supplements to reduce inflammation, diabetes management, hypertension, heart disease, and even cancer prevention [10].	
**Dhonya Pata (Coriandrum sativum)**	Coriandrum sativum, commonly known as Dhonya Pata, is widely found in Bangladesh, particularly in moist or wet areas. It is commonly used in cooking for its fragrance aroma. This plant has many herbal uses, including coronary and peripheral artery diseases, rheumatic, cerebrovascular, and congenital heart disease, in addition to deep vein thrombosis and pulmonary embolism [11].	
**Datura (Datura stramonium)**	Datura is a popular herb and commonly found in woods. Especially in China they have been using it since the early Song Dynasty, Traditional Chinese medicine was used to treat rheumatism, infantile convulsions, asthma, cough, epigastric pain, and surgical anesthetic. This plant also works as an herbal remedy for the flu, common cold, and diarrhea [12].	

This dataset fills the gap of the existing dataset that usually focuses on plants leaves or lacks variations within the dataset. Some existing medicinal datasets include ‘BDMediLeaves,’ [13] which provides 2029 raw images across ten classes. However, this dataset only includes plant leaves, whereas our dataset encompasses both leaf and plant bodies for improved plant recognition. Another dataset, “Bangladeshi Medicinal Plant Classification” [14], contains 5000 images of ten medicinal plants, focusing solely on the plant leaves.

Both of these dataset images are taken in artificial conditions, inside a room, and lack a real-world view of field-sourced images, whereas the ‘BDHerbalPlants’ dataset offers images of diverse conditions within fields. Though there are few overlaps in the medicinal plants, we have considered diverse scenarios when taking the images. Our dataset also includes plants of different sizes and variants, especially for giant plants like neem, which feature both small and large plant images. This will help in object detection tasks and identify the plants from real-world conditions and seamlessly integrate with IoT devices, making it possible to classify these plants in real time.

## 4 Experimental Design, Materials and Methods

### 4.1 Experimental design

**Fig. 2:**
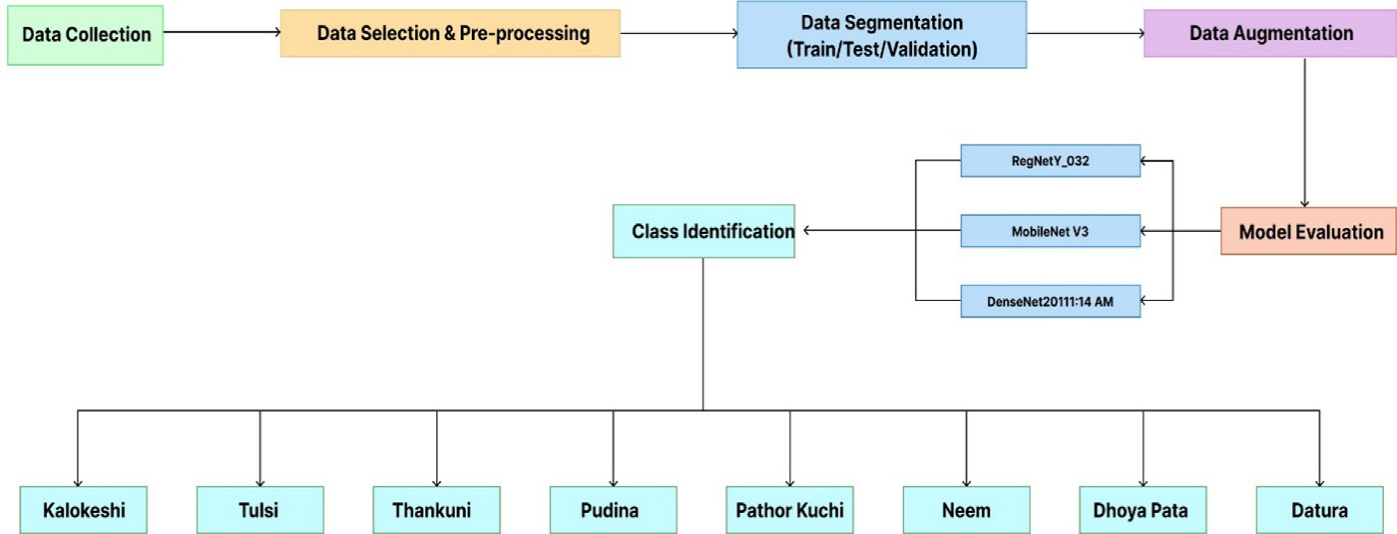
Experimental design workflow for detection and classification of herbal plants.

Herbal plants play a significant role in both traditional and modern medicine. Accurately identifying these plants can be a complex and labor-intensive process. So, computer-aided ways of classifying herbal plants not only increase efficiency but also improve accuracy margin. To create this dataset, considerable effort was invested in capturing various herbal plant species of different sizes and under varying lighting conditions. All 1792 raw data were collected from 260 distinct plants, and all eight classes contain about 200 data each. After collecting data, data was selected, and the raw data was split into three subsets: train, test, and validation. Later, data was pre-processed carefully with necessary steps like resizing, normalizing, color adjustment, etc. The data was augmented with a wide variety of methods. After careful consideration and expert advice, this data has been applied to three DL pre-trained models and ensured that the data quality is good as we verify the results. The detailed workflow of this experiment design is shown in Fig. 2. The figure begins with data collection and outlines all the steps all the way leading up to model classification.

### 4.2 Materials (Camera specification)

Data have been collected maintaining high quality images of herbal using smartphone cameras. Images are collected by Poco F5 and Google Pixel 7 smartphones camera. Almost all the pictures have 4624 × 3472/ 3472 × 4624 pixels or 4080 × 3072 pixels. POCO F5 with 64MP (f/1.8, (wide), 1/2.0″, 0.7 µm) and Google Pixel 7 with 50 MP (f/1.85, 25 mm (wide), 1/1.31″, 1.2 µm) was used in this data collection process.

### 4.3 Image pre-processing

The pre-processing part includes resizing to 512 × 512 pixels to standardize input dimensions. Normalization of pixels is done to enhance convergence during training (scaling to [0, 1]), random brightness adjustment for under‐exposed samples, and contrast enhancement to sharpen feature boundaries for better classification. Additionally, hue and saturation adjustments are made for colour variation between samples. The data is split into training, testing, and validation sets with a 70:20:10 using random sampling. No explicit stratification technique was used. The programmed splitting procedure guarantees that each class remains well-represented in all three splits.

### 4.4 Augmentation

**Fig. 3:**
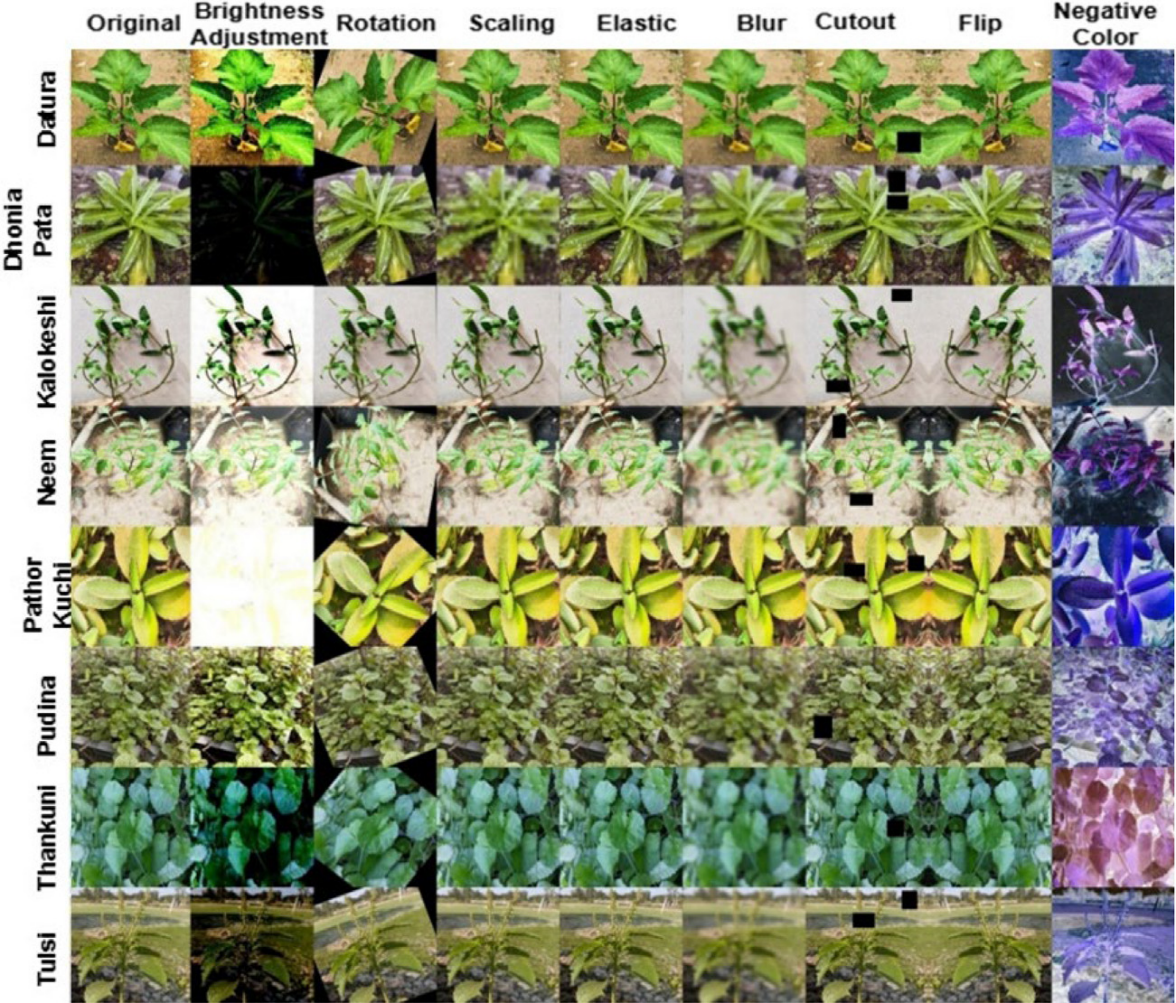
BDHerbalPlants original against augmentated images.

Augmentation techniques were applied to get 1 to 8 augmented data using OpenCV (cv2), NumPy, Albumentations, and PyTorch. This include Rotation (±30 degrees), Scaling (range ±20 %), Elastic Transformation (Alpha: 1.0, Sigma: 50), Gaussian Blur (Blur kernel size: 3 to 7), Brightness Adjustment (brightness limit: ±0.1), Contrast Adjustment (Contrast limit: ±0.1), Coarse Dropout (Max holes: 8, Max size: 50 × 50 pixels), Horizontal Flip (50 % probability), Vertical Flip (20 % probability). Augmentation is done througly using all the techniques and combining together, and a sample result for individual techniques are shown in Fig. 3.

### 4.5 Model performance

To verify the data usability on various ML and DL models, this work has shown train results from three popular models these are, EfficientNetV2_S, Xception and RegNetY (032). The detail information of model training can be seen in Table 3.

**Table 3:** Raw data train and validation result.

Model name	Precision	Recall	Accuracy (raw data)	F1 score (raw data)	Accuracy (augmented data)	F1 score (augmented data)
Xception	0.9846	0.9829	0.9840	0.9832	0.9994	0.9974
DenseNet201	0.9952	0.9950	0.9947	0.9951	0.9939	0.9926
RegNetY032	0.9795	0.9789	0.9786	0.9788	0.9827	0.9827

In all models, accuracy ranges between 97 and 99 % in Raw and is 98 to 99 % in augmented data. Among all the models, DenseNet201 is the best performing model, 99 % accuracy.

Verification of images is necessary to establish authenticity, integrity and quality of images. In several disciplines, these techniques are important in the sense that, photographs are not manipulated in any way or they are not misrepresented. Objective measurement of quality is commonly used in image processing. Key statistical measures include the Structural Similarity Index Metric (SSIM), Peak Signal-to-Noise Ratio (PSNR), Mean Squared Error (MSE), and Root Mean Squared Error (RMSE), along with frequency parameters such as spectral phase and magnitude distortions. Table 4 presents the exceptionally high scores for all ten randomly selected photographs. This indicates that higher verification scores contribute to improved research quality.

**Table 4:** Image verification scores.

Name	Image	SSIM	PSNR	RMSE	MSE
Range	_	(−1 to 1)	(0 to 30)	(0 to ∞)	(0 to ∞)
Preferred Range	_	Close to 1	20 to 30	Close to 0	Close to 0
Datura stramonium		0.74	28.09	41.61	1731.81
Azadirachta indica		0.78	28.11	34.89	1217.35
Coriandrum sativum		0.77	27.95	36.1	1303.13
Mentha arvensis		0.82	27.98	28.58	816.64
Ocimum tenuiflorum		0.8	27.81	31.04	963.2
Centella asiatica		0.84	28.21	30.65	939.12
Kalanchoe pinnata		0.83	27.91	33.21	1102.94
Eclipta prostrata		0.76	27.94	36.42	1326.22

## Limitations

The primary limitation of this dataset is its relatively small number of classes. It is common to see that locating and collecting data on various herbal plants is a very time-consuming task. Even if there are some plants available within some regions the quantity and the season might not match the requirement. Given more time, it is possible to include more of the herbal plant species to overcome this single limitation.

## Ethics Statement

This article has been completed strictly following ethical principles. There has been no harm done to any human or animal during this research. Only the images of plants have been taken by camera. The author has read and followed all the ethical requirements for publication in Data in Brief, making sure it involves no human subject or animal and none of the data has been taken from any social media.

## CRediT authorship contribution statement

**Sunzil Khandaker:** Conceptualization, Methodology, Data curation, Writing – original draft, Validation, Visualization. **Md Mizanur Rahman:** Supervision, Writing – review & editing.

## Data Availability

Mendeley DataBDHerbalPlants (Original data).
